# Personnel protection strategy for healthcare workers in Wuhan during the COVID-19 epidemic

**DOI:** 10.1093/pcmedi/pbaa024

**Published:** 2020-07-20

**Authors:** Fan Fan Hou, Fuling Zhou, Xin Xu, Daowen Wang, Gang Xu, Tao Jiang, Sheng Nie, Xiaoyan Wu, Chanjun Ren, Guangyu Wang, Johnson Yiu-Nam Lau, Xinghuan Wang, Kang Zhang

**Affiliations:** Nanfang Hospital, Southern Medical University; National Clinical Research Center for Kidney Disease; State Key Laboratory of Organ Failure Research, Guangzhou 510515, China; Zhongnan Hospital of Wuhan University, Wuhan 430000, China; Nanfang Hospital, Southern Medical University; National Clinical Research Center for Kidney Disease; State Key Laboratory of Organ Failure Research, Guangzhou 510515, China; Tongji Hospital, Huazhong University of Science and Technology, Wuhan 430030, China; Tongji Hospital, Huazhong University of Science and Technology, Wuhan 430030, China; Jingzhou Central Hospital, Jingzhou 434020, China; Nanfang Hospital, Southern Medical University; National Clinical Research Center for Kidney Disease; State Key Laboratory of Organ Failure Research, Guangzhou 510515, China; Zhongnan Hospital of Wuhan University, Wuhan 430000, China; Wuhan Kingmed Medical Laboratory, Wuhan, China; Department of Computer Science and Technology, Tsinghua University, Beijing 100084, China; Department of Applied Biology and Chemical Technology, Hong Kong Polytechnic University, Hung Hom, Hong Kong, China; Zhongnan Hospital of Wuhan University, Wuhan 430000, China; Center for Biomedicine and Innovations, Faculty of Medicine, Macau University of Science and Technology, Macau, China

**Keywords:** COVID-19, healthcare workers, personal protective equipment (PPE)

## Abstract

**Objective:**

To identify the effectiveness of a personnel protection strategy in protection of healthcare workers from SARS-CoV-2 infection.

**Design:**

During the COVID-19 pandemic, 943 healthcare staff sent from Guangzhou to Wuhan to care for patients with suspected/confirmed COVID-19 received infection precaution training before their mission and were equipped with Level 2/3 personal protective equipment (PPE), in accordance with guidelines from the National Health Commission of China. We conducted a serological survey on the cumulative attack rate of SARS-CoV-2 among the healthcare workers sent to Wuhan and compared the seropositive rate to that in local healthcare workers from Wuhan and Jingzhou.

**Results:**

Serial tests for SARS-CoV-2 RNA and tests for SARS-CoV-2 immunoglobulin M and G after the 6-8 week mission revealed a zero cumulative attack rate. Among the local healthcare workers in Wuhan and Jingzhou of Hubei Province, 2.5% (113 out of 4495) and 0.32% (10 out of 3091) had RT-PCR confirmed COVID-19, respectively. The seropositivity for SARS-CoV-2 antibodies (IgG, IgM, or both IgG/IgM positive) was 3.4% (53 out of 1571) in local healthcare workers from Wuhan with Level 2/3 PPE working in isolation areas and 5.4% (126 out of 2336) in healthcare staff with Level 1 PPE working in non-isolation medical areas, respectively.

**Conclusions and relevance:**

Our study confirmed that adequate training/PPE can protect medical personnel against SARS-CoV-2.

## Introduction

The novel coronavirus SARS-CoV-2, associate disease, COVID-19, has evolved as a major pandemic in less than three months because of the highly infectious nature of the virus and the current intensive social interaction which favors transmission of the virus. Such an explosive pandemic has created unprecedented stress on the healthcare system globally. Protecting healthcare workers is critical for functioning of the system and to prevent the workers serving as a vector for disease transmission.

Although China is a major supplier of personal protective equipment (PPE), the impact of COVID-19 initially created a critical shortage of PPE.^1^ The National Health Commission of China has previously issued technical guidance for prevention of airborne transmission diseases in healthcare facilities with three hierarchical levels of personal protection in 2017, which were further updated for prevention of the spread of COVID-19 in February 2020.^2,3^ A Chinese expert panel also reported a consensus on personal protection in medical institutions during the COVID-19 epidemic.^4^

The efficacy of such measures, however, was never tested in a real pandemic situation until COVID-19. During the pandemic, the sudden surge in demand for healthcare called for unprecedented initiatives. China was building new temporary hospitals within 10 days and a large number of healthcare workers were called to Wuhan. Here, we describe the logistics behind some of the personnel efforts and whether or not the personnel protection strategy was effective.

## Methods

### Study design and participants

We enrolled a total of 8529 healthcare workers, including medical teams aiding Hubei, local healthcare workers in Wuhan and Jingzhou of Hubei Province. Employees in the participating hospitals, including those without direct patient care responsibilities, were invited to take a serological test for antibodies against SARS-CoV-2 and to submit a self-report of gender, age, division, occupation, history of confirmed COVID-19, and history of working in the isolation area for COVID-19 management . The serologic survey was performed between 20 March and 15 April 2020. The Medical Ethics Committee of Nanfang Hospital approved the study and all participants signed the consent form.

### Classification of working area

Healthcare workers were classified into three groups according to their working areas during the epidemic. Members of medical teams aiding Hubei as well as local healthcare workers who had a self-reported history of working in the isolation medical area for COVID-19 management were classified as working in the isolation medical area. Healthcare workers who did not work in the isolation medical area but were directly involved in patient care (physicians, nurses, and technical staff) or those potentially exposed to infectious materials (sanitary workers, staff in the laundry/disinfection facilities) were classified as working in the non-isolation medical area. Healthcare workers without direct patient care responsibility nor exposure to infectious material under the hospital settings (clerical staff or executives) were classified as working in the non-medical area.

### Determination of PPE level

Use of PPE for the healthcare workers was determined by their working area according to the protection guidelines issued by the National Health Commission of China (Table 1). In brief, Level 1 protection is required for healthcare workers working in routine or emergency patient care. PPE for Level 1 protection includes disposable caps, surgical masks, white coats, and hand hygiene. N95/FFP (filtering facepiece, FFP), isolation gowns, and disposable gloves are used when necessary. Level 2 protection is required for healthcare workers who need to enter the isolation medical areas where patients with suspected or confirmed infection are managed. In addition to PPE for Level 1 protection, goggles and full-face shields, long sleeved, fluid repellent gowns, and shoe covers are used. For healthcare workers engaged in aerosol-generating procedures or management of bio-samples from patients with infection, Level 3 protection, including full face shields, eye protection, FFP masks, gloves, and fluid repellent sleeved gowns, is required. Positive pressure helmets can be used when necessary.

**Table 1.** National Health Commission of China Issued Guidelines for Personal Protection in Medical Institutions during COVID-19.^2^, ^3^

Protection Level	Clinical situation	Disposable cap	Surgical mask	N95/FFP mask	Goggles/face shields	Positive pressure helmet	White coat	Isolation gown	Disposable coverall	Disposable gloves	Disposable shoe covers	Hand hygiene (use of sanitizers)
**1**	Routine out- or in-patient clinic, emergency or acute admission unit, hemodialysis, regular operation room, gastrointestinal endoscope room, logistical support	●	●	○			●	○		○		●
**2**	Designated centers (or areas) where patients with confirmed or suspected COVID-19 are managed	●		●	●		●		●	●	●	●
**3**	Aerosol-generating procedures or management of bio-samples from patients with COVID-19	●		●	●	○	●	○	●	●	●	●

● must use, ○ use when necessary.

### Laboratory measurements

Serum samples were collected at local hospitals. All samples were inactivated at 56 °C for 30 min and stored at −20 °C before testing. The antibodies against SARS-CoV-2 were measured at local hospitals using one of the commercialized assay kits approved by the National Medical Products Administration of China. According to the manufacturers, the sensitivity of the assay kits ranged from 87.3% to 94.3%, and the specificity from 99.5% to 100%.

### Statistical analysis

The seropositive rate of the healthcare workers was expressed as a percentage and the corresponding confidence interval was calculated from binomial probabilities using Wilson's method. For healthcare workers working in the non-isolation medical area, seropositive rates stratified by region and division were also estimated, and the top five divisions ranked by the lower boundary of the estimate were listed.

## Results

### Study population

We conducted a serological survey on the cumulative attack rate of SARS-CoV-2 in 8529 healthcare workers in Hubei Province, of which 943 were sent from Guangzhou to Wuhan to care for patients with suspected/confirmed COVID-19; 4495 were local healthcare workers from Wuhan, the epicenter in China, and 3091 were from Jingzhou of Hubei Province, a city 200 km west of Wuhan. Among the healthcare workers, 71% were female and the median age was 33 years (Table 2).

**Table 2.** Seropositive rates among healthcare workers from different regions.

Regions	Total, N	Median age, year	Male, %	RT-PCR positive, N	Seropositive, N	Seropositive rate, % (95% CI)
**Hubei Province**						
Wuhan	4495	33	30.1	113	205	4.6 (4.0, 5.2)
Jingzhou^a^	3091	35	28.9	10	41	1.3 (1.0, 1.8)
**Medical team aiding Hubei**	943	33	38.0	0	0	0.0 (0.0, 0.4)

a A city 200 km from Wuhan.

### Seropositive rate in healthcare workers

All 943 healthcare workers from Guangzhou who were sent to assist Wuhan to combat COVID-19, tested negative for all four reverse transcription polymerase chain reaction (RT-PCR) performed on days 1, 2, 7, and 14. All also tested seronegative for both IgG and IgM for SARS-CoV-2 (10–11 days after they had contact with COVID-19 patients/contacts) (Table 2).

In contrast, among the local healthcare workers in Wuhan and Jingzhou of Hubei Province, 2.5% (113 out of 4495) and 0.32% (10 out of 3091) had RT-PCR confirmed COVID-19, respectively. The seropositivity for SARS-CoV-2 antibodies (IgG, IgM, or both IgG/IgM positive) was 3.4% (53/1571) in local healthcare workers from Wuhan with Level 2/3 PPE working in isolation areas and 5.4% (126/2336) in healthcare staff with Level 1 PPE working in non-isolation medical areas, respectively (Table   3). Similar analysis for the Jingzhou healthcare workers identified seropositivity of 0.3% for those working in the isolation area with Level 2/3 PPE and 1.6% for those working in the non-isolation areas with Level 1 PPE. Note that for those staff who did not provide direct medical services (including sanitary workers, laundry/disinfection staff, elevator operators), 4.4% of the Wuhan healthcare workers and 1.0% of the Jingzhou area were antibody-seropositive, respectively (Table 3). For Wuhan, the top five divisions with the highest estimated cumulative attack rate based on antibody-seropositivity were the hemodialysis unit (12/96, 12.5%), emergency department (6/40, 15%), endoscopy area (9/80, 11.3%), surgery department (40/586, 6.8%), and sanitary department (12/154, 7.8%) (Table   4).

**Table 3.** Seropositive rates for IgG/IgM against SARS-CoV-2 among healthcare workers and the level of PPE used.

Working area	PPE level^a^	Wuhan, Hubei Province			Jingzhou^d^, Hubei Province			Medical team from Guangzhou City coming to support Wuhan		
		Total	Positive	Positive rate, % (95% CI)	Total	Positive	Positive rate, % (95% CI)	Total	Positive	Positive rate, % (95% CI)
Isolation medical area^b^	2 or 3	1571	53	3.4 (2.6, 4.4)	301	1	0.3 (0.0, 1.9)	943	0	0.0 (0.0, 0.4)
Non-isolation medical area^c^	1	2336	126	5.4 (4.5, 6.4)	2168	34	1.6 (1.1, 2.2)			
Non-medical area	0	588	26	4.4 (3.0, 6.4)	622	6	1.0 (0.4, 2.1)			

a PPE level was determined by the working area and protection guidance issued by the National Health Commission of China.

b With confirmed or suspected COVID-19 patients.

c Without confirmed or suspected COVID-19 patients.

d Jingzhou city is approximately 200 km away from Wuhan city and both cities are in Hubei Province.

**Table 4.** Top five divisions with the highest estimated cumulative attack rate based on antibody-seropositivity rate.^a^, ^b^

Division	Total, N	Seropositive, N	Seropositive rate, % (95% CI)
**Wuhan, Hubei**			
Hemodialysis	96	12	12.5 (7.3, 20.6)
Emergency room	40	6	15.0 (7.1, 29.1)
Endoscopy	80	9	11.3 (6.0, 20.0)
Surgery department	586	40	6.8 (5.1, 9.2)
Sanitary department^c^	154	12	7.8 (4.5, 13.1)
**Jingzhou, Hubei**			
Sanitary department^c^	204	5	2.5 (1.1, 5.6)
Surgery department	538	10	1.9 (1.0, 3.4)
Oncology	86	2	2.3 (0.6, 8.1)
Cardiology	91	2	2.2 (0.6, 7.7)
Ophthalmology/otorhinolaryngology	99	2	2.0 (0.6, 7.1)

a Ranked by the lower boundary of estimated seropositive rate.

b Only including healthcare workers in non-isolation medical area.

c Including sanitary workers, staff in laundry/disinfection facilities.

## Discussion

To the best of our knowledge, this is the largest serological survey on the accumulative rate of SARS-CoV-2 infection and the effectiveness of PPE use in healthcare workers. The healthcare staff sent from Guangzhou to Wuhan received infection precaution training before their mission and were equipped with Level 2/3 PPE. Serial tests for SARS-CoV-2 RNA and tests for SARS-CoV-2 immunoglobulin M and G after the 6–8 week mission revealed a zero cumulative attack rate, confirming that adequate training/PPE can protect medical personnel against SARS-CoV-2.

Table 3 summarizes the guideline issued by the National Health Commission of China for personal protection in medical institutions during COVID-19.^2,3^ Note that this guideline was mainly designed for airborne transmitted pathogens and attention was focused on aerosol and contact transmission. Also note that Level 3 differs from Level 2 with the addition of an isolation gown on top of the disposable coverall and potential use of a positive pressure helmet. In the Wuhan situation, positive pressure helmets were generally not used.

Healthcare workers in Wuhan city and the nearby Jingzhou city (around 200 km away from Wuhan, both are in Hubei Province) were updated regularly on the latest recommendations for their protection. The challenge in analysis of data from this group was that these healthcare workers could acquire the virus via patients/staff in the hospital but also through community transmission when not at work.

For healthcare workers coming from outside Hubei Province whose primary role was to engage in direct patient care and clinical management patients with suspected or confirmed COVID-19, an additional strategy was adopted for protection. For this study, our aim was to evaluate the clinical outcomes of this group. First, these workers were recruited, debriefed on the situation in Wuhan, and written consent was obtained from them to participate as part of a medical team in the major hospitals in Wuhan to assist in combating the epidemic. Second, they were given personal protection training and as all would be summoned to care for patients with suspected/confirmed COVID-19, they were all provided with Level 2 or 3 protection (but no positive pressure helmets). Third, it was arranged for all the workers to stay in designated hotels in which only medical staff were accommodated, and all were informed to practice social distancing, limit their exposure to the local community, and wear face masks whenever possible. Finally, there was also a medical team to monitor the mental status of these healthcare workers.

With the epidemic under control around 6–8 weeks after their deployment, the healthcare workers from outside Hubei underwent the following procedures before heading home: around 4–5 days prior to leaving Wuhan, they stopped working in the hospitals, ceased patient contact, and participated in a debriefing period, both to receive information on the next phase but also to give their input on how to improve the system. They were requested to wear face masks whenever possible. There were 943 healthcare workers sent to support Wuhan from hospitals located in Guangzhou. When they came back to Guangzhou (on 20 March 2020), they were required to undergo 14 days of quarantine. They all had SARS-CoV-2 nucleic acid RT-PCR tests performed four times (upon arrival, and on day 2, day 7, and day 14) and serology for SARS-CoV-2 immunoglobulin (IgG and IgM) performed on day 6 (or 10 days after they stopped seeing patients or working in hospital in Wuhan). These results were then compared with data from local healthcare workers from Wuhan and Jingzhou of Hubei Province. All RT-PCR and serology tests were performed in government-approved laboratories using protocols approved by the Chinese FDA as previously described.^5^

This study identifies two important points. First, prior training on use of Level 2/3 PPE, in conjunction with standard infection control practice, was very effective in protecting healthcare personnel from SARS-CoV-2 infection even though they were in direct contact with patients and were actively involved in management of patients with confirmed/suspected COVID-19. This is in striking contrast to previous observations that PPE did not effectively protect healthcare workers from infection during the 2003 SARS outbreak.^6^, ^7^ One possible explanation is that the previous SARS incidence had created a high alert and that current adherence to the personal protection protocol makes the difference.

The second point was the relatively high seropositive rate for SARS-CoV-2 antibodies among the local healthcare workers with Level 1 protection and those working in the non-medical area with no PPE. It is worth re-examining the need for additional training and PPE support for healthcare staff working in non-isolation medical areas, and even non-medical areas with an epidemic of an airborne highly infectious pathogen, especially if PPE supply is not limited.

One potential limitation of this study was that enrollment of subjects for this study was based on voluntary participation (apart from the 943 medical staff from Guangzhou which was mandatory), thus there might be potential bias in the volunteering participants being more eager to observe the rules. Even with this limitation, the personnel protection strategy, coupled together with appropriate coaching and practice, was shown to protect the healthcare personnel sent from Guangzhou to Wuhan with zero cumulative attack rate in this SARS-CoV-2 epidemic. However, there is room for improvement in terms of staff with Level 1 protection working in non-isolated areas and staff in non-medical areas. These data provide a framework to assist other countries that are still in the midst of combating this pandemic, and could be used to prepare for future epidemics/pandemics.

## Conclusions

Our study confirmed that adequate training and PPE can protect medical personnel against SARS-CoV-2 infection.
